# Area-level factors influencing geographical distribution of myopia prevalence among school-aged children and adolescents in Northwest China

**DOI:** 10.7189/jogh.15.04144

**Published:** 2025-06-13

**Authors:** Bixia Wei, Yanli Qin, Kaili Wu, Huiqin Wang, Lin Ding, Tao Shen

**Affiliations:** 1 People’s Hospital of Xinjiang Uygur Autonomous Region, Urumqi, China; 2 State Key Laboratory of Ophthalmology, Zhongshan Ophthalmic Center, Sun Yat-sen University, Guangdong Provincial Key Laboratory of Ophthalmology and Visual Science, Guangdong Provincial Clinical Research Center for Ocular Diseases, Guangzhou, China

## Abstract

**Background:**

Northwest China is an ideal spot to investigate geographic differences in myopia prevalence due to the region’s unequal allocation of socioeconomic and geo-environmental variables. Yet, the overall geographic distribution of myopia in the region remains unclear.

**Methods:**

We conducted a school-based, cross-sectional prevalence survey of myopia in all 14 prefectures of Xinjiang, China. We employed Moran's *I* index to quantify the spatial distribution of myopia prevalence. We conducted correlation and multivariate logistic regression analyses to determine the independent association of socioeconomic and geo-environmental factors with myopia.

**Results:**

We included a total of 64 277 Han (36.6%), Uygur (38.1%), and other ethnic groups (25.3%) children and adolescents aged 6–18 years. The overall myopia prevalence was 52.2%, with northern areas (56.9%) showing a substantially greater prevalence than southern areas (42.2%). Myopia prevalence varies widely between regions, ranging from 65.1% (Karamay, located in northern Xinjiang) to 24.1% (Hotan in southern Xinjiang), and a spatial aggregation of myopia prevalence was identified (Moran's *I =* 0.364; *P* = 0.090; Z-score = 1.696). Rather than variations in latitude, the geographical distribution of myopia prevalence may be influenced by disparities in area-level socioeconomic factors (*e.g.* economic income and health care coverage) and geo-environmental factors (*e.g.* sunshine duration and environmental greening).

**Conclusions:**

There are notable differences in the prevalence of myopia in children and adolescents between northern and southern Xinjiang of northwest China, where socioeconomic circumstances and geo-environmental factors differ greatly. The influencing factors identified in our study may be key area-level intervention targets for myopia control in public health policy.

Myopia has become a significant global public health concern in recent decades. The prevalence of myopia and high myopia is projected to undergo a substantial increase in the upcoming decades, with myopia potentially affecting 50% and high myopia 10% of the global population by 2050 [1]. Myopia prevalence exhibits significant regional variations worldwide, with particularly elevated rates observed in East Asian countries [2–4]. Myopia among children and adolescents in China also displays considerable heterogeneity based on geographical location due to China's vast territory encompassing diverse latitudes, longitudes, and altitudes [5]. Notably, a higher prevalence of myopia has been reported among children and adolescents in the eastern coastal and northern regions of China [4,6]. However, recent studies have revealed that Northwest China does not exhibit low myopia prevalence either [7,8]. In addition, similar inter-regional differences in myopia prevalence exist within Northwest China, which has never been studied in detail and may be related to the interaction of multiple factors.

The high or rapidly increasing prevalence of myopia in a specific area contradicts the notion that genetic factors primarily determine it [9,10]. While inter-generational or inter-regional genetic differences are slow and minimal, socioeconomic and geo-environmental factors can occur much more quickly and significantly impact myopia development. Education and limited time spent outdoors have been identified as major risk factors for myopia [11]. Previous studies have established a close association between various socioeconomic factors and myopia at both individual levels (*e.g.* education, family income, lifestyle) and area levels (*e.g.* gross domestic product (GDP), population density, hospital bed density, health care coverage) [12–14]. Similarly, environmental factors play an important role in myopia development and can be categorised into individual-level (*e.g.* outdoor activities, near-work tasks, smoking habits, diet patterns, housing conditions) and area-level (*e.g.* sunshine duration, air quality, green space availability, geographical location) factors [15–18]. Individual-level factors have been extensively studied as independent influencing factors for myopia. In contrast, few studies have investigated the relationships between these area-level factors and myopia [6,12,18,19], especially in explaining its prevalence disparities in different geographical distributions. Moreover, considering the presence of various confounding variables such as demographic differences, socioeconomic status, and environmental influences, investigating influencing factors at the area level would provide valuable insights into identifying risk factors associated with myopia.

Xinjiang Uygur autonomous region, which encompasses one-sixth of China's total land area, is situated in the northwest. The Tianshan Mountains divide Xinjiang into northern and southern regions, each characterised by distinct geo-environments, economic levels, climatic conditions, and living habits. Hence, Xinjiang presents an ideal setting to investigate the geographical disparities in myopia prevalence due to its unique geographic peculiarities. In comparison with national trends, school-aged children and adolescents in Xinjiang exhibit a slightly lower prevalence of myopia than those in southeastern regions of China [6,8]. With a diverse population comprising mainly Han and Uygur ethnic groups, Xinjiang demonstrates notable variations in myopia prevalence across different ethnicities [7]. However, the overall geographical distribution of myopia and its influencing factors remain unknown within Xinjiang. It was hypothesised that the variation in myopia prevalence between different prefectures of Xinjiang is attributable to specific area-level factors. Therefore, we aim to explore regional differences in myopia distribution among children and adolescents aged 6–18, a critical period for myopia development, within the distinctive northwest region of China. Additionally, we seek to examine the impact of area-level socioeconomic and geo-environmental factors on myopia prevalence from a public health perspective.

## METHODS

### Study design and participants

We conducted a school-based cross-sectional study from January to June 2023 in Xinjiang, China, to screen the myopia in children and adolescents aged 6–18. We used a multistage stratified random cluster sampling method for myopia screening in 14 prefectures of Xinjiang, including nine prefectures (Urumqi, Karamay, Turpan, Hami, Changji, Bortala, Ili, Altay, and Tacheng) in northern Xinjiang and five prefectures (Bayingolin, Kezilesu, Aksu, Kashi, and Hotan) in southern Xinjiang, administratively. We selected two screening sites in each prefecture, and screened both urban and rural areas for myopia at each site. We excluded students with remarkable ocular or systemic disorders. We obtained a written informed consent form from the parents or their guardians. We collected detailed demographic data of participants from schools, including gender, ethnicity, place of birth, date of birth, grade level, and past medical history.

### Refractive examination

All participants underwent an ophthalmic evaluation based on a standard protocol for common ocular diseases by trained ophthalmologists. We measured non-cycloplegic refraction with an autorefractor (ARK-1, Nidek Co, Ltd) instead of cycloplegic refraction, due to logistical constraints or ethical considerations. We performed the refractive examination three times for each eye and averaged the results. If the difference between multiple measurements of the same eye was >0.50 diopters (D), we repeated the refraction measurement. We used the spherical equivalent (SE) of the refractive error, defined as the spherical value of refractive error plus one-half of the cylindrical value, to classify participants into non-myopia, low myopia, moderate myopia, and high myopia. We defined myopia as SE≤−0.50 D, and subdivided it into low myopia (SE = −3.00, −0.50 D), moderate myopia (SE = −6.00, −3.00 D), and high myopia (SE≤−6.00 D). We calculated the intraclass correlation coefficient (ICC) to evaluate the interocular correlation of the SE. Since the SE was highly consistent in both eyes (ICC = 0.924), we only used the data from the right eye for statistical analysis.

### Exposure data

We retrieved socioeconomic data from the Statistics Bureau of Xinjiang Uygur Autonomous Region [20], including per capita GDP, per capita disposable income, population density, doctors per 1000 people, hospital beds per 1000 people, and health care coverage. We sourced geo-environmental data, including year-round sunshine duration, annual average temperature, annual air quality index (AQI), urban per capita green space, normalised difference vegetation index (NDVI), and latitude, from the National Geomatics Center of China [21], the Global Change Research Data Publishing & Repository [22], and the Resource and Environmental Science Data Platform [23]. Considering the influence lag on myopia prevalence by exposure factors, we selected all the exposure data from 2020, three years before myopia screening (Table S1 in the **Online Supplementary Document**).

### Statistical analysis

We estimated the sample size using the formula n = u_α_^2^ × p × (1 − p) / δ^2^, considering myopia prevalence of *P* = 32%, u_α_ = 1.96, δ = 0.05, *P* = 0.32, n = 1.96^2^ × 0.32 × 0.68 / 0.05^2^ ≈ 334. We estimated the loss to follow-up rate as 10%, and the stratified cluster sampling efficiency was 1.5, n = 334 / 0.9 × 1.5 ≈ 557. We stratified the survey into 14 prefectures, three school levels (primary, junior middle, and senior middle), and two residential areas (urban and rural). Therefore, we calculated our target sample size as follows: N = n ×14 × 3 × 2 = 46 788. Based on the actual screening results, we included 64 277 individuals.

We estimated the prevalence of myopia for the overall sample and then stratified by age, gender, school stage, ethnicity, and area. To assess the geographical distribution of myopia prevalence in Xinjiang, we complied a map of myopia prevalence in 14 prefectures using ArcMap, version 10.7 (Environmental Systems Research Institute, Redland, Canada, USA). In addition, we analysed the overall distribution patterns of myopia prevalence through global spatial autocorrelation, and calculated the global Moran’s *I* index to describe the spatial distribution features.

We adopted Pearson χ^2^ tests to evaluate the differences between different groups in myopia and high myopia prevalence. We used an independent student *t* test and one-way ANOVA to compare the differences in SE between different groups. We tested the normality of the distributions using Kolmogorov-Smirnov and Shapiro-Wilk tests. We performed Pearson correlation analyses to assess the potential risk factors, and used multivariate logistic regression models to investigate the independent association between risk factors and myopia. We used the variance inflation factor to detect high correlations among predictors, and included all the potential confounding factors as covariates in the multivariate logistic regression model except for those with a high level of collinearity (*i.e.* per capita GDP, population density, annual average temperature, and urban per capita green space). We included *P* < 0.05 in the multivariable logistic regression model by the backwards selection method, and determined odds ratios (ORs) and 95% confidence intervals (CIs). We used SPSS, version 25.0 (SPSS, Chicago, Illinois, USA) for all statistical analyses, and considered a two-tailed *P*-value of <0.05 statistically significant.

## RESULTS

### Overall characteristics of study participants

This study included a total of 64 277 students aged 6–18 from primary schools, junior middle schools, and senior middle schools across 14 prefectures in Xinjiang. The mean age of the overall participants was 12.13 years (standard deviation (SD) = 3.39), of whom 48.9% were males. The mean SE was −1.16 D (SD = 2.02), and the overall prevalence of myopia was 52.2% (95% CI = 51.8–52.6), and high myopia was 3.2% (95% CI = 3.0–3.3). The myopic status and myopia prevalence increased with age in both males and females, and more than half of the participants over the age of 11 had myopia (Figure S1 and Table S2 in the **Online Supplementary Document**). A notable gender-related disparity emerges at the age of eight, with a higher prevalence of myopia observed in females compared to males (Table S3 in the **Online Supplementary Document**). The females and Han students had lower SE than males and Uygur students (*P* < 0.001) (Figure S2, Panel A), and myopia prevalence was higher in females and Han students than in males and Uygur students (*P* < 0.001) (Figure S2, Panel B).

### Geographical distribution of myopia

**Figure 1:**
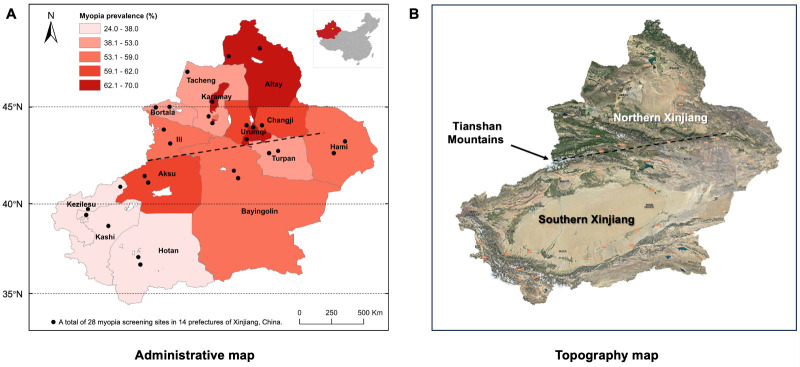
Distribution of myopia prevalence in 14 prefectures of Xinjiang. **Panel A.** Distribution map of myopia prevalence in 14 prefectures of Xinjiang. **Panel B.** Satellite map shows significant geomorphological differences between northern and southern Xinjiang.

The refractive status and myopia prevalence had significant regional differences (Table S3 in the **Online Supplementary Document**). The urban area had a myopia prevalence of 54.5% (95% CI = 54.0–54.9), which was significantly higher than in the rural area (47.5%; 95% CI = 47.0–47.9) (*P* < 0.001) (Figure S2, Panel B). In addition, the northern area had a myopia prevalence of 56.9% (95% CI = 56.6–57.2), which was significantly higher than in the southern area (42.2%, 95% CI = 41.5–42.9) (*P* < 0.001). The myopia prevalence of each prefecture in Xinjiang varied from 65.1% in Karamy located in northern Xinjiang to 24.1% in Hotan located in southern Xinjiang (Figure S2, **Table 1**). Only four of 14 prefectures had myopia prevalence lower than 50%, three fourths of which were in southern Xinjiang. On the other hand, eight out of nine northern prefectures had a myopia prevalence of over 50%, except for Turpan with a myopia prevalence of 39.6%. The administrative map of Xinjiang illustrates the geographical distribution of myopia prevalence in the 14 prefectures (**Figure 1**, Panel A). The global spatial autocorrelation analysis indicated that the distribution of myopia prevalence was clustered rather than randomly distributed (Moran’s *I =* 0.364; *P* = 0.090; Z-score = 1.696). The clustered regions with a high myopia prevalence were in northern Xinjiang, such as Karamay, Altay, and Urumqi. In addition, the distributions of myopia prevalence in females (Moran’s *I =* 0.382; *P* = 0.074; Z-score = 1.790) and rural areas (Moran’s *I =* 0.549; *P* = 0.009; Z-score = 2.628) were found to be relatively clustered in the stratified global spatial autocorrelation analysis (Table S4 in the **Online Supplementary Document**).

**Table 1:** Refractive status and myopia prevalence in 14 prefectures of Xinjiang (n = 64 277)

				SE (in D), x̄ (SD)				Prevalence of myopia, %				Prevalence of high myopia, %			
	**Cases, n (%)**	**Gender ratio (male/female)**	**Age (in years), x̄ (SD)**	**Total**	**Male**	**Female**	***P*-value**	**Total**	**Male**	**Female**	***P*-value**	**Total**	**Male**	**Female**	***P*-value**
**Northern prefectures**															
Altay	3897 (6.1)	1904/1993	12.27 (3.40)	−1.47 (2.00)	−1.24 (1.96)	−1.69 (2.00)	<0.001	62.7	56.5	68.6	<0.001	3.5	2.8	4.2	0.020
Tacheng	4147 (6.5)	2087/2060	12.33 (3.45)	−1.19 (1.79)	−0.99 (1.72)	−1.38 (1.84)	<0.001	52.8	47.0	58.6	<0.001	2.3	1.8	2.9	0.026
Bortala	3820 (5.9)	1905/1915	12.18 (3.38)	−1.02 (2.10)	−0.90 (2.01)	−1.13 (2.19)	<0.001	51.0	47.1	54.8	<0.001	3.0	2.7	3.2	0.406
Karamay	3908 (6.1)	2078/1830	12.17 (3.42)	−1.71 (2.12)	−1.69 (2.18)	−1.72 (2.05)	0.629	65.1	63.4	67.1	0.015	4.8	5.0	4.5	0.537
Changji	4250 (6.6)	2156/2094	11.79 (3.31)	−1.50 (2.07)	−1.37 (2.01)	−1.63 (2.13)	<0.001	61.6	57.8	65.4	<0.001	3.2	3.2	3.2	0.999
Urumqi	11 320 (17.6)	5690/5630	12.59 (3.28)	−1.62 (2.31)	−1.52 (2.29)	−1.72 (2.34)	<0.001	62.3	60.1	64.6	<0.001	5.5	5.6	5.3	0.439
Ili	4064 (6.3)	1932/2132	12.50 (3.36)	−1.17 (2.03)	−0.96 (1.88)	−1.35 (2.14)	<0.001	53.1	47.5	58.2	<0.001	2.8	2.4	3.2	0.101
Hami	4178 (6.5)	2133/2045	12.29 (3.48)	−1.25 (1.99)	−1.12 (1.85)	−1.38 (2.11)	<0.001	54.7	51.6	58.0	<0.001	2.8	2.2	3.4	0.017
Turpan	4214 (6.6)	1976/2238	12.44 (3.50)	−0.65 (1.61)	−0.56 (1.51)	−0.74 (1.69)	<0.001	39.6	37.0	41.9	0.001	1.8	1.5	2.0	0.269
**Southern prefectures**															
Bayingolin	4298 (6.7)	2032/2266	12.14 (3.40)	−1.46 (2.11)	−1.29 (2.10)	−1.61 (2.11)	<0.001	58.8	55.0	62.2	<0.001	4.1	3.8	4.5	0.273
Aksu	4208 (6.5)	2098/2110	12.16 (3.37)	−1.32 (1.95)	−1.25 (1.96)	−1.39 (1.93)	0.016	59.8	56.9	62.7	<0.001	3.3	3.2	3.4	0.757
Kezilesu	3288 (5.1)	1584/1704	12.29 (3.34)	−0.55 (1.31)	−0.43 (1.23)	−0.66 (1.37)	<0.001	37.4	31.4	43.1	<0.001	1.0	0.6	1.3	0.054
Kashi	4547 (7.1)	2110/2437	12.01 (3.49)	−0.59 (1.46)	−0.53 (1.45)	−0.64 (1.46)	0.011	30.1	26.1	33.5	<0.001	1.3	1.4	1.2	0.574
Hotan	4138 (6.4)	1778/2360	12.65 (3.29)	+0.05 (1.65)	+0.12 (1.49)	−0.01 (1.76)	0.013	24.1	22.9	25.0	0.134	0.6	0.3	0.8	0.075

D – diopter, SD – standard deviation, SE – spherical equivalent, x̄ – mean

### Factors associated with prevalent myopia

Various factors, including socioeconomic and geo-environmental factors, were compared between participants with and without myopia to analyse the variables influencing myopia development (Table S5 in the **Online Supplementary Document**). The results indicated significant differences in all involved factors between the two groups (*P* < 0.001). Among the socioeconomic factors, the participants with myopia showed higher per capita GDP, per capita disposable income, population density, and doctors per 1000 people, and lower hospital beds per 1000 people and health care coverage. Among the geo-environmental factors, the participants with myopia showed higher annual air quality, urban per capita green space, NDVI_250m_, and latitude, and lower year-round sunshine duration and annual average temperature.

Univariate logistic regression analysis showed that myopia was associated with all the demographic, socioeconomic, and geo-environmental factors (**Table 2**). In the multivariate logistic regression model, all the demographic factors had strong impacts on myopia, in which older age, female, and Han ethnicity were positively correlated with myopia. Middle school students aged 13–18 were more likely to have myopia than primary school students aged 6–12, with the OR being 3.214 (95% CI = 3.103–3.328). Females were associated with a 37% (OR = 1.373; 95% CI = 1.327–1.421) increase in the relative risk of myopia, and Uygur Chinese were associated with 69% (OR = 0.309; 95% CI = 0.296–0.324) decrease in the relative risk of myopia compared with Han Chinese.

**Table 2:** Logistic regression analysis for factors associated with myopia

		Univariate		Multivariate*	
	**Myopia, n (%)**	**OR (95% CI)**	***P*-value**	**OR (95% CI)**	***P*-value**
**Demographic factors**					
Age (in years)					
*6–12*	13 115 (39.9)	ref		ref	
*13–18*	20 433 (65.0)	2.797 (2.709–2.888)	<0.001	3.214 (3.103–3.328)	<0.001
Gender					
*Male*	15 451 (49.1)	ref		ref	
*Female*	18 097 (55.2)	1.274 (1.235–1.314)	<0.001	1.373 (1.327–1.421)	<0.001
Ethnicity					
*Han*	15 746 (66.9)	ref		ref	
*Uygur*	9090 (27.1)	0.291 (0.280–0.302)	<0.001	0.309 (0.296–0.324)	<0.001
*Others*	8712 (53.6)	0.571 (0.548–0.595)	<0.001	0.530 (0.505–0.556)	<0.001
**Socioeconomic factors**					
Per capita GDP (1000 CNY)					
*<60*	12 380 (43.7)	ref			
*≥60*	21 168 (58.9)	1.852 (1.794–1.911)	<0.001		
Per capita disposable income (in CNY)					
*<33 000*	13 266 (43.9)	ref		ref	
*≥33 000*	19 125 (59.2)	1.849 (1.791–1.908)	<0.001	1.058 (1.012–1.107)	0.013
Population density (/km^2^)					
*<20*	15 291 (47.8)	ref			
*≥20*	18 257 (56.5)	1.419 (1.376–1.464)	<0.001		
Doctors per 1000 people					
*<2*	14 653 (44.5)	ref		ref	
*≥2*	18 895 (60.2)	1.886 (1.828–1.946)	<0.001	1.145 (1.073–1.222)	<0.001
Hospital beds per 1000 people					
*<6*	20 369 (55.5)	ref		ref	
*≥6*	13 179 (47.8)	0.733 (0.710–0.756)	<0.001	0.631 (0.596–0.669)	<0.001
Healthcare coverage (%)					
*<75*	21 168 (58.9)	ref		ref	
*≥75*	12 380 (43.7)	0.540 (0.523–0.557)	<0.001	0.840 (0.800–0.882)	<0.001
**Geo-environmental factors**					
Year-round sunshine duration (hours)					
*<2800*	19 471 (54.1)	ref		ref	
*≥2800*	14 077 (49.7)	0.839 (0.813–0.865)	<0.001	0.596 (0.563–0.630)	<0.001
Annual average temperature (in °C)					
*<10*	18 796 (60.0)	ref			
*≥10*	14 752 (44.8)	0.542 (0.525–0.559)	<0.001		
Annual air quality (%)					
*<80*	19 979 (49.6)	ref		ref	
*≥80*	13 569 (56.5)	1.319 (1.277–1.362)	<0.001	0.969 (0.912–1.029)	0.300
Urban per capita green space (in m^2^)					
*<15*	14 704 (54.0)	ref			
*≥15*	18 844 (50.9)	0.884 (0.857–0.912)	<0.001		
NDVI_250m_					
*<0.19*	19 179 (55.4)	ref		ref	
*≥0.19*	14 369 (48.5)	0.757 (0.734–0.781)	<0.001	0.784 (0.744–0.826)	<0.001
Latitude (in °N)					
*<43*	12 594 (43.6)	ref		ref	
*≥43*	20 954 (59.2)	1.874 (1.816–1.934)	<0.001	1.021 (0.969–1.075)	0.439

°C – degrees centigrade, °N – degrees north, CI – confidence interval, GDP – gross domestic product, NDVI – normalised difference vegetation index, OR – odds ratio, ref – reference

*The variance inflation factor (VIF) was calculated during multivariate logistic regression analysis, and collinearity was considered present if VIF>10. So, the factors with VIF>10 (per capita GDP, population density, annual average temperature, and urban per capita green space) were excluded from the multivariate logistic regression model.

**Figure 2:**
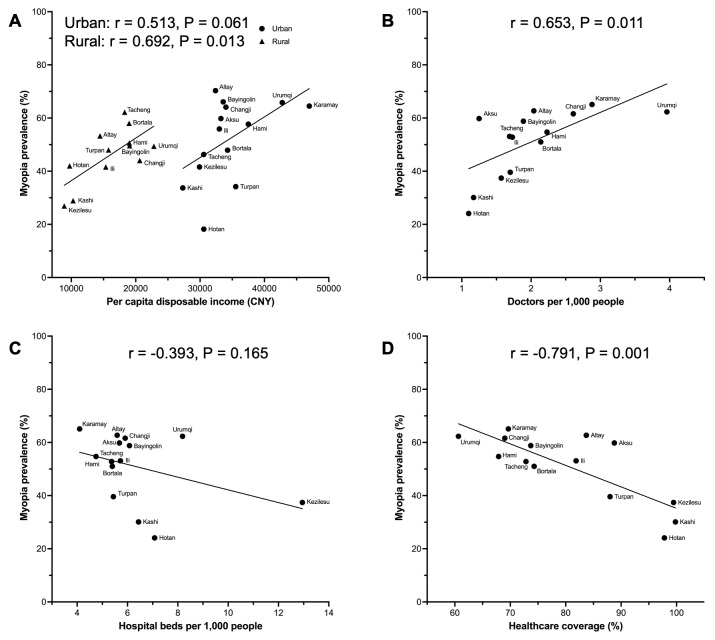
Scatter plots of correlations between socioeconomic factors and myopia prevalence. **Panel A.** Per capita disposable income. **Panel B.** Doctors per 1000 people. **Panel C.** Hospital beds per 1000 people. **Panel D.** Healthcare coverage.

As for the socioeconomic factors, higher per capita disposable income, higher doctors per 1000 people, lower hospital beds per 1000 people, and lower health care coverage were positively correlated with myopia (**Figure 2**, **Table 2**). The participants with the number of hospital beds per 1000 people over 6 (OR = 0.631; 95% CI = 0.596–0.669) and health care coverage over 75% (OR = 0.840; 95% CI = 0.800–0.882) were less likely to have myopia.

**Figure 3:**
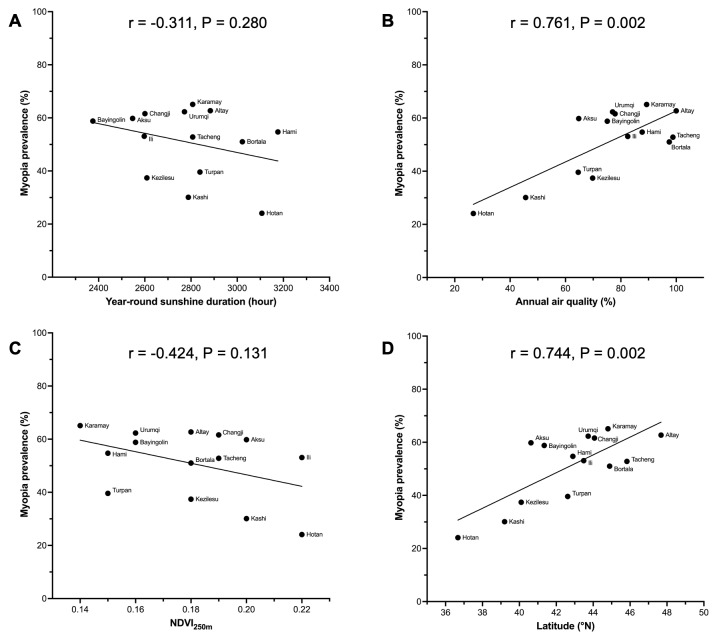
Scatter plots of correlations between geo-environmental factors and myopia prevalence. **Panel A.** Year-round sunshine duration. **Panel B.** Annual air quality. **Panel C.** NDVI_250m_. **Panel D.** Latitude. NDVI – normalised difference vegetation index, °N – degrees north

Among the geo-environmental factors, only the longer year-round sunshine duration and higher NDVI_250m_ were negatively correlated with myopia (**Figure 3**, **Table 2**). The lower likelihoods of having myopia were found in the participants with year-round sunshine duration over 2800 hours (OR = 0.596; 95% CI = 0.563–0.630) and NDVI_250m_ over 0.19 (OR = 0.784; 95% CI = 0.744–0.826). Although the annual air quality (*r* = 0.761; *P* = 0.002) and latitude (r = 0.744; *P* = 0.002) had strong correlations with myopia prevalence, neither air quality (*P* = 0.300) nor latitude (*P* = 0.439) reached statistical significance as predictors in the multivariate logistic regression model.

## DISCUSSION

### Geographic distribution of myopia prevalence

The present study indicated that the prevalence of myopia among children and adolescents aged 6–18 in Xinjiang was 52.2%, which is consistent with the findings of previous studies conducted in this region [7,8,24]. Nevertheless, while previous studies have merely indicated discrepancies in the prevalence of myopia across diverse ethnic groups [7,8], our research is the first to illustrate marked regional variations in the prevalence of myopia between northern and southern Xinjiang. Of the 14 prefectures, the three with the highest prevalence were in northern Xinjiang with a latitude higher than 43° north (N). In comparison, the three with the lowest prevalence were in southern Xinjiang with a latitude lower than 43° N. However, the global spatial autocorrelation analysis showed that the spatial clustering of myopia prevalence is not statistically significant at the conventional 0.05 threshold (Moran’s *I =* 0.364; *P* = 0.090; Z-score = 1.696), and the potential reasons for this include sample size, spatial scale, and number of prefectures. The sunshine duration has mainly been used in previous research to explain variations in the prevalence of myopia at various latitudes. Previous studies have reported a high prevalence of myopia at high latitudes, which has been attributed to the limited sunshine duration at these latitudes [25]. Conversely, other research has found that the prevalence of myopia is lower in northern China with high latitudes because of the longer sunshine duration [18]. Additionally, it has been observed that the prevalence of myopia is relatively low in Norway, a country situated at a high latitude where sunshine duration is limited [17]. These findings were consistent with the current study in that a high prevalence of myopia was not associated with latitude. Still, the phenomenon cannot be attributed to sunshine duration alone.

With an area of 1.66 million km^2^, Xinjiang is the largest provincial-level administrative region in China in terms of land area, spanning more than 1500 km and 15 degrees of latitude from north to south, and nearly 2000 km from east to west, encompassing five time zones. In addition to the latitudinal disparities, the northern and southern Xinjiang, separated by the Tianshan Mountains, exhibit pronounced contrasts in geo-environments, climatic conditions, and economic levels (**Figure 1**, Panel B). The northern Xinjiang has expansive grasslands and fertile valleys, experiencing a temperate continental climate. In contrast, southern Xinjiang is dominated by the Taklamakan Desert, which gives rise to an arid desert climate. The northern region of Xinjiang is economically more developed than the southern region. It has been demonstrated that regional differences between northern and southern Xinjiang, encompassing socioeconomic and geo-environmental factors, are associated with the prevalence of myopia in other geographical regions. After adjusting for confounding variables, the multivariate regression analysis demonstrated that socioeconomic factors (*e.g.* economic income and health care coverage) and geo-environment factors (*e.g.* sunshine duration and environmental greening) exert a more pronounced influence on the prevalence of myopia than geographic latitude.

### Socioeconomic factors

In China, regions with elevated economic advancement (*i.e.* higher per capita GDP or per capita income) have demonstrated a heightened prevalence of myopia [5,26], which is consistent with our study. One potential explanation is that individuals in more economically developed regions face heightened educational demands due to greater competitiveness [11]. The association between individual-level education and myopia has been extensively investigated [13,27]. An additional potential explanation is that economic development may alter individual-level lifestyles and behaviours, including reducing outdoor activities and increasing near-work activity. These individual-level changes have been identified as significant contributing factors to myopia [28,29]. Although a higher GDP per capita was previously linked to a higher prevalence of high myopia, this variable was not identified as a significant predictor of the condition in the present study [30]. Higher population density appears to be an independent risk factor for myopia [6,14], but our study suggested it is not associated with myopia.

With regard to medical security factors, it has been established that the correlation between the number of hospital beds per 1000 people and myopia represents a protective factor [6,26]. No previous study reported the relationship between myopia prevalence and the number of doctors per 1000 people or health care coverage. Although our finding indicated that doctors per 1000 people are a risk factor for myopia, it should be noted that our analysis focussed on the total number of doctors, rather than the number of ophthalmologists. Therefore, it does not accurately reflect the level of ophthalmic medical coverage. It can be reasonably deduced that regions with a more significant number of doctors are more likely to be characterised by a higher GDP, which is a recognised risk factor for myopia. Consequently, regions with more doctors are influenced by risk factors such as area-level GDP and income, they contribute less to eye care coverage.

### Geo-environmental factors

The longer sunshine duration has been identified as a major protective factor against myopia [18,31,32]. The regions of Xinjiang enjoy an adequate level of sunshine, with an annual duration of 2500–3000 hours. In our study, a 40% reduction in the prevalence of myopia was observed in areas with an annual sunshine duration exceeding 2800 hours. Previous studies have indicated that increased exposure to sunshine, particularly in the form of time spent outdoors, can play a role in the prevention of myopia progression [33,34]. Furthermore, a correlation has been identified between poorer sunshine and an increased prevalence of myopia, with this association being particularly pronounced among primary school-aged children [35]. As mentioned earlier, a positive correlation between annual sunshine duration and the prevalence of myopia has been found in some areas [17], and one possible explanation is that the influence of environmental factors is less pronounced than that of human activities [6].

Environmental greening was assessed in terms of both NDVI and urban per capita green space in this study, where NDVI may be more applicable to accurately assessing the degree of environmental greening that has an impact on myopia [16]. The presence of a well-arranged green space, comprising larger areas, enhanced connectivity, increased aggregation, reduced fragmentation, and a shorter distance between patches, was found to be correlated with a slower progression in the prevalence of myopia [36]. Our results indicated that if the area-level NDVI increased to 0.19 or more, the myopia prevalence would be reduced by approximately 22%. Although southern Xinjiang in general is mostly desert, the areas where the population lives permanently are oases in the desert, so the sites where we conducted our myopia screening were no less green environments. For example, Hotan is mostly deserted with snow-capped mountains, but the urban and rural areas have the highest NDVI of all the areas surveyed. Whereas the high NDVI at this site reflects the fact that there is more green space accessible to children when they are outdoors. Numerous studies have adequately demonstrated that NDVI is associated with the prevalence of myopia to varying degrees and that the varying degrees of influence are related to the different NDVI parameters studied [16,37–40]. Per capita green space has also been studied as an indicator of environmental greening in the prevalence of myopia; however, as our findings suggested, its correlation with myopia was not as strong as NDVI [6,36,41,42].

Exposure to ambient air pollution significantly impacts myopia development, but the detailed mechanisms by which air pollutants interfere with myopia remain unclear [43]. Some studies suggested that direct exposure to air pollutants might be associated with a higher risk of visual impairment through increasing oxidative stress and inflammation around the eyes [39]. There is a positive correlation between exposure to high concentrations of particulate matter or nitrogen oxides and the prevalence of myopia [44,45]. The AQI is a widely used comprehensive indicator of overall air pollution levels based on multiple air pollutants, including particulate matter and nitrogen oxides. However, no studies have hitherto reported the relationship between AQI and myopia. Thus, we employed AQI as a research indicator to assess the relationship between area-level air pollution and myopia prevalence. The results of our study unexpectedly revealed a significant positive correlation between annual AQI and myopia prevalence. Hotan, situated in southern Xinjiang, is characterised by some of China's most severe air pollution [46]. However, our study revealed that it exhibits the lowest prevalence of myopia among the 14 prefectures in Xinjiang. Nevertheless, the multivariate regression analysis model showed that annual AQI was not statistically associated with myopia (*P* = 0.300). The AQI is associated with various socioeconomic and geo-environmental factors, including GDP, population density, NDVI, annual temperature, and sunshine duration [456]. Consequently, despite the existence of numerous proposed mechanisms by which air pollution may influence the onset of myopia, including peripheral hyperopia defocus, the dopamine pathway, and retinal ischaemia [43], our findings suggest that a straightforward causal relationship between myopia and AQI may not be a universal phenomenon.

Despite fluctuations in ambient temperature between northern and southern Xinjiang, our findings indicated that this variable is not associated with myopia when other potential confounding factors are considered. Previous studies have similarly not identified a relationship between ambient temperature and myopia occurrence [18].

The prevalence of myopia in rural areas was more influenced by geo-environmental factors between northern and southern Xinjiang and was more likely to be aggregated (Moran’s *I* = 0.549; *P* = 0.009; Z-score = 2.628). One possible explanation is that these geo-environmental factors are more potent than socioeconomic factors and that several confounding factors have complicated the relationship. Rural areas in Northwest China are virtually unaffected by socioeconomic factors, and there is little difference between northern and southern Xinjiang in socioeconomic factors. This result might suggest that geo-environmental factors have a more significant influence on the prevalence of myopia than socioeconomic factors. Previous studies have shown that myopia is more prevalent among urban girls and children, although the relative rise has been fastest in rural areas [5].

The prevalence of myopia is known to vary by ethnicity [47], and our results were similar to previous findings in Xinjiang [7,8]. The higher prevalence of myopia among the Han population is likely attributable to a combination of genetic susceptibility, heightened educational demands, reduced outdoor activity, dietary differences, and lifestyle changes associated with urbanisation. These factors interact synergistically, resulting in a heightened susceptibility to developing myopia among the *Han* population compared to the Uygur population. We also found a significant increase in the prevalence of myopia from the age of eight, with differences between males and females, and a stabilisation in myopia from the age of 15, similar to the results of previous studies [48]. Interventions aimed at modifying the various factors that influence the prevalence of myopia should therefore begin before the age of 15 at the latest, and preferably by the age of 8, for example, by encouraging children and adolescents to bathe in full sunlight and by improving the greening of school and home environments.

The strength of our study lies in the large amount of representative data obtained from a region-wide survey, which comprehensively analysed the socioeconomic and geo-environmental factors affecting the distribution of myopia prevalence in Xinjiang, a region of Northwest China with special geographic characteristics, and provided new ideas for the prevention and control of myopia. However, our study has some limitations. First, school-aged children had less than one D myopic SE difference using non-cycloplegic *vs.* cycloplegic refraction [49]. The use of non-cycloplegic refraction in this study may have overestimated the prevalence of myopia. Second, because of the cross-sectional design, it was impossible to determine the temporal causality of the area-level factors on myopia. Moreover, our analyses using one-year data from three years ago did not analyse the longer-term effects of the area-level factors on myopia, and the onset of myopia may be a cumulative effect of the multi-year impact of these factors. Regarding the area-level factors, the evidence from longitudinal studies is insufficient to prove causality. Therefore, prospective cohort studies on these area-level risk factors should be conducted to provide further evidence on whether they are responsible for the onset of myopia. Third, although there are no reports of altitude differences associated with the prevalence of myopia, there are significant altitude differences across Xinjiang, and our study did not examine the effect of altitude. Fourth, although we attempt to control confounding factors, the potential impact of unmeasured confounders is not adequately addressed. For example, factors such as parental education or occupational exposure may influence myopia risk but are not considered in detail. This gap could lead to biased associations and limit the reliability of the study.

## CONCLUSIONS

This is the first study showing notable regional differences in the prevalence of myopia in school-aged children and adolescents between northern and southern Xinjiang in Northwest China. The prevalence of myopia showed a clear clustering of high prevalence in northern Xinjiang and low prevalence in southern Xinjiang. Various factors influence the onset and development of myopia, and current research has demonstrated that both socioeconomic and geo-environmental factors are associated with myopia. Differences in the spatial distribution of myopia prevalence were caused by socioeconomic factors (*e.g.* economic income and health care) and geo-environmental factors (*e.g.* sunshine duration and environmental greening) at the area level. The present study provided more specific findings at the area level than previous research on individual-level factors. Controlling multiple confounding factors, the above factors may be more critical in myopia development. This highlights the need to define the exposures responsible for the rapid increase in myopia prevalence, as modifiable socioeconomic and geo-environmental factors may provide an essential basis for preventive interventions. For example, improving accessible health care coverage, optimising school-based outdoor programs, and increasing environmental greening in urban planning are tools that have the potential to reduce regional myopia prevalence effectively. Our study provides new perspectives for the comprehensive prevention and management of myopia.

## Additional Material


Online Supplementary Document
