# Supplementary material for: Area-level factors influencing geographical distribution of myopia prevalence among school-aged children and adolescents in Northwest China
Source: J Glob Health. 2025 Jun 13;15:04144. doi: 10.7189/jogh.15.04144 (PMC12161485; doi:10.7189/jogh.15.04144)
Supplement: Online Supplementary Document [file jogh-15-04144-s001.pdf]

**Supplement to: Wei B, Qin Y, Wu K, Wang H, Ding L, Shen T. Area-level factors influencing geographical distribution of myopia prevalence among school-aged children and adolescents in Northwest China. J Glob Health. 2025;15:04144.**

**Figure S1. Age-gender distribution pyramid of myopia prevalence**

A graphical illustration showing the age-gender distribution of myopia prevalence, which increased with age in both males and females and remained stable at over 60% after the age of 15. The myopia prevalence in males and females of each age was marked inside the bars.

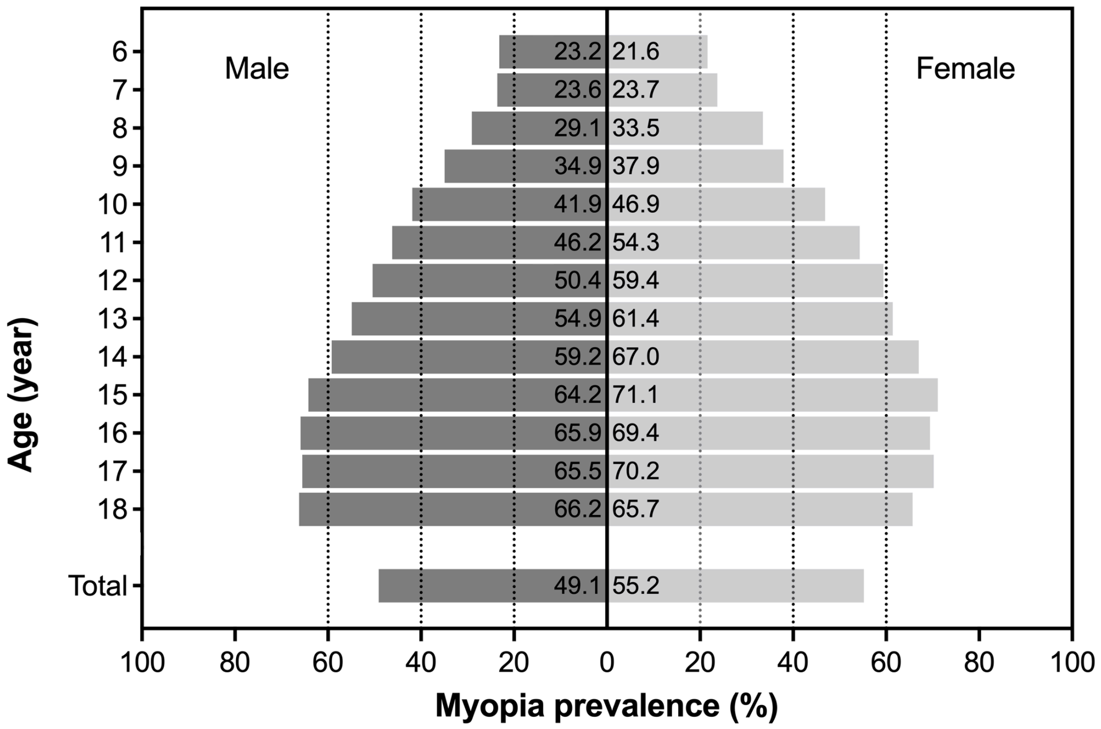


**Figure S2. Stratified comparative analysis of refractive status and myopia prevalence**

A. Comparative analysis of SE stratified by gender, ethnicity, residence, and geographical area.

B. Comparative analysis of myopia prevalence stratified by gender, ethnicity, residence, and geographical area.

The females and Han students showed lower SE and higher myopia prevalence than males and Uygur students (P<0.001). In addition, urban and northern areas also showed lower SE and higher myopia prevalence than rural and southern areas

(P<0.001). There were statistically significant differences in SE and myopia prevalence between the two ethnic groups (P<0.05). SE = spherical equivalent; D = diopter.

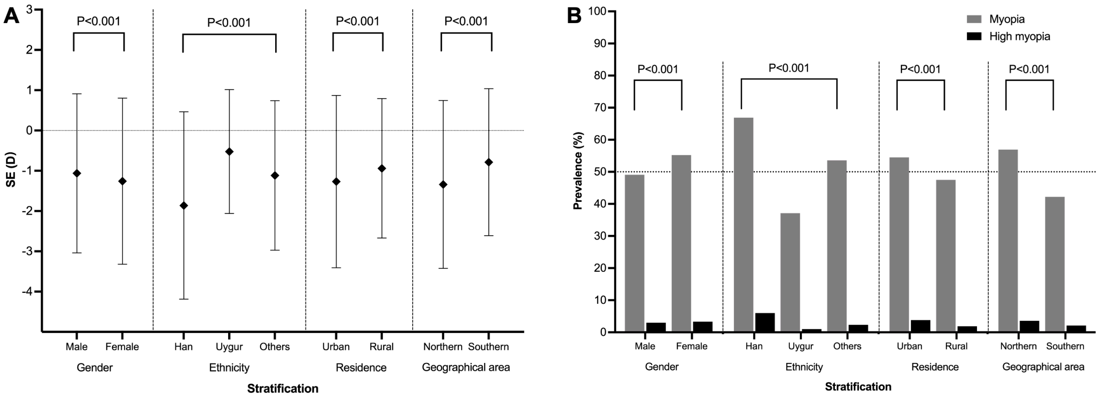


**Figure S3. Myopia prevalence in 14 prefectures of Xinjiang**

Ten of 14 prefectures had a prevalence of myopia over 50% and a prevalence of moderate to high myopia over 10%. Karamay, Altay, and Urumqi ranked the top three prefectures with the highest myopia prevalence among school-aged children and adolescents in Xinjiang, with a myopia prevalence of 65.1%, 62.7%, and 62.3%, respectively. Prefectures with higher myopia prevalence were mainly located in northern Xinjiang. Hotan, Kashi, and Kezilesu, all of which are located in southern Xinjiang, had the lowest myopia prevalence of 24.1%, 30.1%, and 39.6%, respectively. Five underlined prefectures are located in the southern Xinjiang.

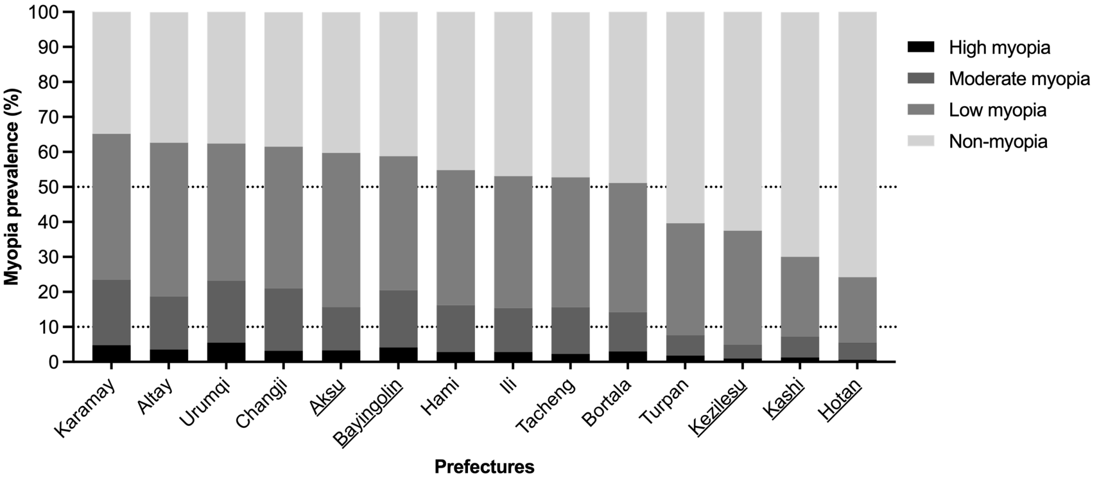

**Table S1. Area-level socio-economic and geo-environmental information of 14 prefectures in 2020**

| | Prefecture | Socio-economic factors | | | | | | | | | Geo-environmental factors | | | | | |
|---|---|---|---|---|---|---|---|---|---|---|---|---|---|---|---|---|
| | | Total GDP (billion CNY) | Population size (thousand) | Per capita GDP (thousand CNY) | Urban per capita disposable income (CNY) | Rural per capita disposable income (CNY) | Population density (/km$^2$) | Doctors per 1,000 people | Hospital beds per 1,000 people | Healthcare coverage (%) | Year-round sunshine duration [a] (hour) | Annual average temperature [a] (°C) | Annual air quality [a, b] (%) | Urban per capita green space [a] (m$^2$) | NDVI$_{250m}$ | Latitude [a] (°N) |
| Northern | Altay | 335 | 669 | 55.45 | 32404 | 14461 | 5.67 | 2.04 | 5.59 | 83.72 | 2883.6 | 5.5 | 100.0 | 24.97 | 0.18 | 47.68 |
| | Tacheng | 738 | 1139 | 60.50 | 30570 | 18290 | 10.81 | 1.73 | 5.37 | 72.83 | 2807.4 | 9.2 | 98.8 | 19.47 | 0.19 | 45.83 |
| | Bortala | 377 | 488 | 79.33 | 34276 | 18978 | 17.94 | 2.14 | 5.40 | 74.30 | 3022.5 | 7.8 | 97.5 | 14.25 | 0.18 | 44.89 |
| | Karamay | 887 | 490 | 200.29 | 46963 | - | 63.36 | 2.88 | 4.10 | 69.65 | 2807.4 | 9.2 | 89.3 | 12.77 | 0.14 | 44.80 |
| | Changji | 1387 | 1614 | 86.25 | 34024 | 20640 | 21.96 | 2.61 | 5.91 | 69.02 | 2600.4 | 8.1 | 77.9 | 18.44 | 0.19 | 44.07 |
| | Urumqi | 3337 | 4054 | 94.82 | 42770 | 22827 | 285.17 | 3.96 | 8.19 | 60.65 | 2771.8 | 8.7 | 77.0 | 10.82 | 0.16 | 43.73 |
| | Ili | 1266 | 2848 | 50.99 | 33000 | 15356 | 50.41 | 1.69 | 5.72 | 81.88 | 2597.6 | 10.6 | 82.5 | 9.97 | 0.22 | 43.49 |
| | Hami | 608 | 673 | 97.96 | 37505 | 19022 | 4.74 | 2.23 | 4.75 | 67.89 | 3176.4 | 10.7 | 87.7 | 15.16 | 0.15 | 42.90 |
| | Turpan | 373 | 694 | 55.02 | 35545 | 15781 | 9.96 | 1.70 | 5.44 | 88.00 | 2838.9 | 16.3 | 64.6 | 19.39 | 0.15 | 42.62 |
| Southern | Bayingolin | 1106 | 1614 | 86.48 | 33592 | 19060 | 3.42 | 1.89 | 6.08 | 73.69 | 2375.1 | 12.1 | 75.1 | 15.50 | 0.16 | 41.35 |
| | Aksu | 1315 | 2714 | 51.29 | 33239 | 14588 | 20.67 | 1.25 | 5.68 | 88.78 | 2547.2 | 12.2 | 64.8 | 24.84 | 0.20 | 40.63 |
| | Kezilesu | 169 | 622 | 27.27 | 29918 | 8907 | 8.58 | 1.57 | 12.95 | 99.47 | 2609.0 | 12.6 | 69.8 | 15.19 | 0.18 | 40.10 |
| | Kashi | 1130 | 4496 | 28.40 | 27321 | 10276 | 27.75 | 1.17 | 6.44 | 99.79 | 2788.4 | 14.0 | 45.6 | 17.70 | 0.20 | 39.20 |
| | Hotan | 406 | 2505 | 20.17 | 30586 | 9733 | 10.13 | 1.10 | 7.08 | 97.80 | 3107.0 | 11.4 | 26.7 | 7.21 | 0.22 | 36.67 |

GDP = gross domestic product; CNY = Chinese Yuan; °C = degree centigrade; NDVI = normalized difference vegetation index; °N = degrees north.

[a] Data were collected from major cities in the region.

[b] Proportion of days with excellent and good air quality index (AQI = 0-100).

**Table S2. Refractive status and myopia prevalence in different ages (N = 64,277)**

| Age | Cases | | Gender ratio | SE (Mean ± SD, D) | | | Prevalence of myopia (%) | | | Prevalence of high myopia (%) | | |
|---|---|---|---|---|---|---|---|---|---|---|---|---|
| (year) | N | % | (male/female) | Total | Male | Female | Total | Male | Female | Total | Male | Female |
| 6 | 1048 | 1.6 | 496/552 | -0.12 ± 1.03 | -0.12 ± 1.05 | -0.12 ± 1.01 | 22.3 | 23.2 | 21.6 | 0.4 | 0.6 | 0.2 |
| 7 | 4679 | 7.3 | 2381/2298 | -0.10 ± 1.13 | -0.10 ± 1.07 | -0.09 ± 1.19 | 23.6 | 23.6 | 23.7 | 0.2 | 0.3 | 0.2 |
| 8 | 5444 | 8.5 | 2825/2619 | -0.32 ± 1.37* | -0.27 ± 1.19 | -0.37 ± 1.55 | 31.2* | 29.1 | 33.5 | 0.4 | 0.2 | 0.6 |
| 9 | 5412 | 8.4 | 2770/2642 | -0.46 ± 1.35* | -0.41 ± 1.37 | -0.51 ± 1.32 | 36.4* | 34.9 | 37.9 | 0.5 | 0.5 | 0.6 |
| 10 | 5373 | 8.4 | 2694/2679 | -0.71 ± 1.58* | -0.64 ± 1.53 | -0.78 ± 1.62 | 44.4* | 41.9 | 46.9 | 0.9 | 0.8 | 1.0 |
| 11 | 5416 | 8.4 | 2733/2683 | -0.93 ± 1.73* | -0.80 ± 1.65 | -1.05 ± 1.80 | 50.2* | 46.2 | 54.3 | 1.2 | 1.1 | 1.3 |
| 12 | 5479 | 8.5 | 2790/2689 | -1.16 ± 1.93* | -1.06 ± 1.92 | -1.27 ± 1.93 | 54.8* | 50.4 | 59.4 | 2.5 | 2.3 | 2.8 |
| 13 | 5790 | 9.0 | 2789/3001 | -1.30 ± 1.90* | -1.19 ± 1.87 | -1.41 ± 1.91 | 58.3* | 54.9 | 61.4 | 2.4 | 2.5 | 2.4 |
| 14 | 5812 | 9.0 | 2906/2906 | -1.56 ± 2.08* | -1.43 ± 2.04 | -1.68 ± 2.12 | 63.1* | 59.2 | 67.0 | 3.8 | 3.6 | 4.0 |
| 15 | 5497 | 8.6 | 2635/2862 | -1.80 ± 2.28* | -1.67 ± 2.26 | -1.94 ± 2.30 | 67.8* | 64.2 | 71.1 | 5.6 | 5.3 | 5.8 |
| 16 | 5578 | 8.7 | 2481/3097 | -1.95 ± 2.36* | -1.88 ± 2.35 | -2.01 ± 2.37 | 67.9* | 65.9 | 69.4 | 6.9 | 6.9 | 6.9 |
| 17 | 5133 | 8.0 | 2298/2835 | -2.04 ± 2.46* | -1.95 ± 2.45 | -2.11 ± 2.47 | 68.1* | 65.5 | 70.2 | 7.6 | 7.6 | 7.5 |
| 18 | 3616 | 5.6 | 1665/1951 | -1.90 ± 2.46* | -2.01 ± 2.56 | -1.80 ± 2.36 | 65.9 | 66.2 | 65.7 | 7.5* | 8.9 | 6.2 |
| Total | 64277 | 100.0 | 31463/32814 | -1.16 ± 2.02* | -1.06 ± 1.98 | -1.26 ± 2.06 | 52.2* | 49.1 | 55.2 | 3.2 | 3.0 | 3.3 |

SE = spherical equivalent; D = diopter; N = number of participants; SD = standard deviation.

* The statistically significant difference between the genders (P<0.05).

**Table S3. Stratified refractive status and myopia prevalence in Xinjiang (N = 64,277)**

| Variables | Cases | | Age | SE | | Prevalence of myopia | | Prevalence of high myopia | |
|---|---|---|---|---|---|---|---|---|---|
| | N | % | (Mean ± SD, year) | Mean ± SD, D | *P*-value | % (95% CI) | *P*-value | % (95% CI) | *P*-value |
| Gender | | | | | | | | | |
| Male | 31463 | 48.9 | 12.17 ± 3.36 | -1.06 ± 1.98 | <0.001 | 49.1 (48.6, 49.7) | <0.001 | 3.0 (2.8, 3.2) | 0.054 |
| Female | 32814 | 51.1 | 12.44 ± 3.41 | -1.26 ± 2.06 | | 55.2 (54.8, 55.5) | | 3.3 (3.2, 3.4) | |
| School stage | | | | | | | | | |
| Primary | 32413 | 50.4 | 9.44 ± 1.81 | -0.58 ± 1.55 | <0.001* | 39.2 (38.8, 39.6) | <0.001* | 0.9 (0.8, 1.0) | <0.001* |
| Junior middle | 17196 | 26.8 | 13.95 ± 1.08 | -1.48 ± 2.05 | | 61.8 (61.4,62.2) | | 3.4 (3.3, 3.6) | |
| Senior middle | 14668 | 22.8 | 16.72 ± 0.94 | -2.08 ± 2.45 | | 69.6 (69.3, 70.0) | | 7.9 (7.6, 8.1) | |
| Ethnicity | | | | | | | | | |
| Han | 23520 | 36.6 | 12.23 ± 3.42 | -1.86 ± 2.33 | <0.001* | 66.9 (66.4, 67.6) | <0.001* | 6.0 (5.7, 6.3) | <0.001* |
| Uygur | 24515 | 38.1 | 12.41 ± 3.41 | -0.52 ± 1.54 | | 37.1 (36.7, 37.5) | | 1.0 (0.9, 1.1) | |
| Others | 16242 | 25.3 | 12.27 ± 3.30 | -1.12 ± 1.86 | | 53.6 (53.2, 54.1) | | 2.3 (2.2, 2.5) | |
| Residence | | | | | | | | | |
| Urban | 43537 | 67.7 | 12.66 ± 3.43 | -1.27 ± 2.14 | <0.001 | 54.5 (54.0, 54.9) | <0.001 | 3.8 (3.6, 3.9) | <0.001 |
| Rural | 20740 | 32.3 | 11.57 ± 3.18 | -0.94 ± 1.73 | | 47.5 (47.0, 47.9) | | 1.9 (1.8, 2.0) | |
| Geographical area | | | | | | | | | |
| Northern | 43798 | 68.1 | 12.24 ± 3.39 | -1.34 ± 2.09 | <0.001 | 56.9 (56.6, 57.2) | <0.001 | 3.6 (3.5, 3.8) | <0.001 |
| Southern | 20479 | 31.9 | 12.34 ± 3.39 | -0.79 ± 1.82 | | 42.2 (41.5, 42.9) | | 2.1 (1.9, 2.3) | |

SE = spherical equivalent; D = diopter; N = number of participants; SD = standard deviation; CI = confidence interval.

* There were statistically significant differences between the two ethnic groups (P<0.05).

**Table S4. Global spatial autocorrelation analysis of myopia prevalence**

| Variables | Global bivariate Moran's *I* | Z score | *P*-value [a] |
|---|---|---|---|
| Overall | 0.364 | 1.696 [b] | 0.090 |
| Gender | | | |
| Male | 0.328 | 1.542 | 0.123 |
| Female | 0.382 | 1.790 [b] | 0.074 |
| School stage | | | |
| Primary | 0.281 | 1.375 | 0.169 |
| Junior middle | 0.306 | 1.457 | 0.145 |
| Senior middle | 0.295 | 1.489 | 0.137 |
| Ethnicity | | | |
| Han | 0.134 | 1.110 | 0.267 |
| Uygur | 0.346 | 1.613 | 0.107 |
| Others | 0.264 | 1.547 | 0.122 |
| Residence | | | |
| Urban | 0.073 | 0.578 | 0.563 |
| Rural | 0.549 | 2.628 [b] | 0.009 |

[a] P values were calculated using the Z test.

[b] Z scores over 1.650 was considered statistically clustered.

**Table S5. Comparison of variables associated with the occurrence of myopia (N = 64,277)**

| Variables | | Myopia | Non-myopia | *P*-value [a] |
|---|---|---|---|---|
| Cases, N | | 33548 | 30729 | - |
| Age (year) | | 13.24 ± 3.12 | 11.29 ± 3.37 | <0.001 |
| Gender, male/female (%) | | 46.10/53.90 | 52.11/47.89 | <0.001 |
| SE (D) | | -2.46 ± 1.94 | 0.26 ± 0.80 | <0.001 |
| Socio-economic factors | Per capita GDP (thousand CNY) | 80.27 ± 41.29 | 66.32 ± 39.23 | <0.001 |
| | per capita disposable income (CNY) | 31155.86 ± 9960.65 | 28155.31 ± 10376.59 | <0.001 |
| | Population density (/km$^2$) | 76.27 ± 109.08 | 55.66 ± 93.30 | <0.001 |
| | Doctors per 1,000 people | 2.32 ± 0.92 | 2.03 ± 0.88 | <0.001 |
| | Hospital beds per 1,000 people | 6.36 ± 1.78 | 6.59 ± 2.01 | <0.001 |
| | Healthcare coverage (%) | 76.14 ± 12.12 | 81.35 ± 13.82 | <0.001 |
| Geo-environmental factors | Year-round sunshine duration (hour) | 2763.80 ± 200.66 | 2796.60 ± 207.28 | <0.001 |
| | Annual average temperature (°C) | 10.03 ± 2.48 | 10.86 ± 2.68 | <0.001 |
| | Annual air quality (%) | 79.43 ± 15.32 | 72.21 ± 21.23 | <0.001 |
| | Urban per capita green space (m$^2$) | 15.06 ± 4.27 | 14.83 ± 4.41 | <0.001 |
| | $NDVI_{250m}$ [b] | 0.18 ± 0.24 | 0.18 ± 0.23 | <0.001 |
| | Latitude (°N) | 43.27 ± 2.42 | 42.28 ± 2.86 | <0.001 |

N = number; D=diopter; GDP = gross domestic product; CNY = Chinese Yuan; °C = degree centigrade; NDVI = normalized difference vegetation index; °N = degrees north.

[a] For categorical variables, a $\chi 2$ test was used; for continuous variables, a t test was used.

[b] $NDVI_{250m}$ for myopia was 0.1822 ± 0.0241, and for non-myopia was 0.1770 ± 0.0231.
